# Openness to Changing Religious Views Is Related to Radial Diffusivity in the Genu of the Corpus Callosum in an Initial Study of Healthy Young Adults

**DOI:** 10.3389/fpsyg.2018.00330

**Published:** 2018-03-27

**Authors:** Jiansong Xu, Clayton H. McClintock, Iris M. Balodis, Lisa Miller, Marc N. Potenza

**Affiliations:** ^1^Department of Psychiatry, Yale School of Medicine, Yale University, New Haven, CT, United States; ^2^Spirituality Mind Body Institute, Department of Counseling and Clinical Psychology, Teachers College, Columbia University, New York City, NY, United States; ^3^Peter Boris Centre for Addictions Research, Department of Psychiatry and Behavioural Neurosciences, Michael G. DeGroote School of Medicine, McMaster University, Hamilton, ON, Canada; ^4^Child Study Center, Yale School of Medicine, Yale University, New Haven, CT, United States; ^5^Department of Neurobiology, Yale School of Medicine, Yale University, New Haven, CT, United States; ^6^Connecticut Mental Health Center, New Haven, CT, United States

**Keywords:** neuroimaging, diffusion tensor imaging, white matter, religion, spirituality

## Abstract

A quest orientation to religion is characterized by a search for answers to complex existential questions, a perception of religious doubt as positive, and an openness to change one’s religious views as one grows and changes. This orientation is inversely related to fundamentalism, authoritarianism, and prejudice and directly related to cognitive complexity, openness to experience, and prosociality. To date, the neural correlates of religious quest have not been investigated. This study assessed the relationships between measures linked to white-matter integrity and quest religious orientation among 24 healthy participants using diffusion tensor imaging (DTI) and the quest scale. A tract-based spatial statistical analysis whole-brain-corrected initially employing an accepted threshold (*p*_TFCE_ < 0.05) and then applying a Bonferroni correction (*p*_TFCE_ < 0.0042) identified a region of the genu of the corpus callosum as showing radial diffusivity measures being related to openness to change religious beliefs. When not employing a Bonferroni correction (*p*_TFCE_ < 0.05), the openness-to-change subscale of the quest scale negatively correlated with radial diffusivity and mean diffusivity measures in extensive white-matter regions in both hemispheres that include the corpus callosum body, genu, and splenium, superior longitudinal fasciculus, forceps minor, external capsule, and inferior fronto-occipital fasciculus. No relationships were found with the other subscales. These findings suggest that a greater openness to change one’s religious views is associated with better white-matter integrity specifically in the genu of the corpus callosum and likely in a more extensive set of white-matter structures interconnecting widespread cortical and subcortical regions in the brain across hemispheres. They, furthermore, suggest structural similarities that may link this tendency to associated positive psychological traits, including creative cognition and post-traumatic growth.

## Introduction

Proposed as a third fundamental approach to religion along with Gordon Allport’s intrinsic and extrinsic religiosity, quest orientation refers to a form of religiosity that embraces honesty, doubt, and openness in the face of existential questions and complex life circumstances (Batson and Schoenrade, 1991). A multidimensional construct, religious quest as captured by the quest scale includes the following domains: (1) a search for answers to complex existential questions, (2) a perception of religious doubt as positive, and (3) an openness to change one’s religious views as one grows and changes. Despite the centrality of this orientation to religion and its association with a number of positive psychological traits, which include prosociality, openness to experience, and post-traumatic growth (Calhoun et al., 2000; Batson et al., 2001; Simpson et al., 2010), little is known about the neural structures that support this type of religiosity. Here, using diffusion tensor imaging (DTI) measures, we studied how white-matter structures related to aspects of religious quest.

Religious quest is inversely related to aspects of close-mindedness that include fundamentalism and authoritarianism (Altemeyer and Hunsberger, 1992; Hunsberger, 1995; Duck and Hunsberger, 1999), which, in turn, may predict prejudice toward those outside one’s own identity group (Altemeyer and Hunsberger, 1992; Brandt and Van Tongeren, 2015). In contrast, studies suggest that quest orientation is related to less prejudice and greater post-conventional reasoning (Batson et al., 1993; Cottone et al., 2007). Moreover, individuals high in religious quest tend to display empathetic and prosocial behavior (Batson et al., 1993, 2001). Quest religious orientation is also associated with the personality trait of openness to experience (Simpson et al., 2010). Those with this orientation tend to have both greater cognitive complexity and preference for cognitive consistency (Batson and Raynor-Prince, 1983; Barrett et al., 2005). Additionally, in the aftermath of traumatic life events, the degree of post-traumatic growth has been found to directly correlate with the quest domain of openness to change religious views (Calhoun et al., 2000).

Though the neural correlates of quest religious tendencies have not been directly investigated, brain imaging studies on potentially similar features, particularly the construct of openness to experience, serve to provide some relevant context. As measured by the Revised NEO Personality Inventory (NEO PI-R; Costa and McCrae, 1992a,b), openness has been observed to positively correlate with activity in the dorsolateral prefrontal cortex (dlPFC) during cognitive tasks and in the orbitofrontal cortex (OFC) during the resting state (DeYoung et al., 2005; Sutin et al., 2009). Openness is also associated with measures suggestive of better white-matter integrity in both hemispheres of the dlPFC, as well as the superior longitudinal fasciculus (SLF), corona radiata (CR), anterior cingulum, forceps minor, corpus callosum body, and regions adjacent to the middle frontal gyrus (MFG) and inferior frontal gyrus (IFG) (Xu and Potenza, 2012). These white-matter regions interconnect many cortical and subcortical regions in the brain. Additionally, the capacity for cognitive flexibility is associated with dlPFC activity and prefrontal measures related to white-matter integrity (Badre and Wagner, 2006; Nyhus and Barceló, 2009). Taken together, these findings suggest that connections bilaterally in the prefrontal cortex (PFC), the corpus callosum, and other cortical and subcortical brain regions may serve as a neural feature underlying religious quest.

Diffusion tensor imaging (DTI) measures water diffusion in multiple directions (Assaf and Pasternak, 2008). It can be used to generate fractional anisotropy (FA) values by calculating the normalized standard deviation of axial (λ_1_) and radial diffusivities (λ*_T_*) to index the degree to which the water diffusion deviates from isotropic diffusion in white matter, and mean diffusivity (MD) by averaging λ_1_ and λ_T_ to index the overall diffusivity (Assaf and Pasternak, 2008; Neil, 2008). FA correlates positively with the ratio of λ_1_ and λ_T_, while MD correlates positively with the sum of λ_1_ and λ_T_. Therefore, FA and MD are sensitive to different patterns of changes in λ_1_ and λ_T_. For example, an increase in both λ_1_ and λ_T_ may significantly increase MD without significantly changing FA, and a decrease in λ_1_ along with an increase in λ_T_ may significantly decrease FA without significantly changing MD. Therefore, both FA and MD are often used as indexes of white-matter integrity, and a decreased FA and/or an increased MD value in the white matter is usually interpreted as reflecting poorer white-matter integrity (Alexander et al., 2007).

This study aimed to investigate in a preliminary fashion the relationships between measures related to white-matter integrity and aspects of quest orientation to religion. Based on findings of openness on the NEO being related to measures associated with better white-matter integrity and the possibility that openness on the NEO and related to the construct of openness to change on the quest scale, we hypothesized that scores on the openness-to-change subscale specifically would be positively correlated with measures related to better white-matter integrity in prefrontal areas, corpus callosum, and other regions that interconnect widespread cortical and subcortical regions across both hemispheres. We explored correlations with the two other subscales of the quest scale as we believe that this would represent a negative control condition that would provide discriminant validity for potential findings related to openness to change religious views.

## Materials and Methods

### Participants

Individuals were recruited by media ads and provided written informed consent to participate in the protocol that was approved by the Yale Human Investigations Committee. Participants were screened using the Structured Clinical Interview (SCID) for Axis I Psychiatric Disorders (First et al., 1995, 1997) and provided urine samples to assess recent use of cocaine, opioids, stimulants, marijuana, and benzodiazepines. Handedness was assessed by asking participants which hand they usually use, and left-handed individuals were excluded. Participants were excluded if any metabolites of these substances were positive in their urine samples. Other exclusionary criteria included pregnancy, current psychiatric diagnoses, or unstable medical conditions. We acquired both DTI and self-reported data relating to aspects of religious quest from 24 English-speaking young adults (9 females), with a mean age of 22.2 years [range: 18–27, standard deviation (SD) = 2.3]. Mean education was 15.0 years (SD = 1.7). Two individuals were of Hispanic ethnicity. Races included white (*n* = 15), black (*n* = 4), Asian (*n* = 3), and other (*n* = 2). One participant was married, while all others were single.

### Quest Scale

The quest scale (Batson and Schoenrade, 1991) is a 12-item measure that assesses perceptions of religion as a quest, conceptualized by the authors as “openly facing complex existential questions… and resisting clear-cut, pat answers” (Batson and Schoenrade, 1991, p. 430). Each item is rated on a nine-point scale ranging from 1 (strongly disagree) to 9 (strongly agree). The scale has been found to have three domains, with scores for which each range from 4 to 36 and the domains relating to: (1) Readiness to Face Existential Questions without Reducing their Complexity (e.g., “I have been driven to ask religious questions out of a growing awareness of the tensions in my world and in my relation to my world”); (2) Self-Criticism and Perception of Religious Doubt as Positive (e.g., “For me, doubting is an important part of what it means to be religious”); and, (3) Openness to Change (e.g., “As I grow and change, I expect my religion also to grow and change”). Reliability for the quest scale is satisfactory, with alpha coefficients ranging from 0.75 to 0.81, and the mean total score in previous samples of young adults ranged from 59.4 to 60.5 (Batson and Schoenrade, 1991).

### Scanning Procedures

DTI data were acquired with a 3.0T Siemens Trio scanner at the Yale Magnetic Resonance Research Center. Diffusion sensitizing gradients were applied along 32 directions using *b* values of 0 (*b*_0_ image) and 1000 s/mm^2^ (TR = 7400, TE = 115, matrix = 128 × 128, FOV = 256 mm × 256 mm). Forty contiguous slices parallel to the AC-PC line were acquired, and each slice was 3.0 mm thick (Xu et al., 2010). Two repetitions were acquired for averaging. A high-resolution T1 image was routinely acquired and examined by a neuroradiologist to identify any structural anomalies.

### Image Processing

The procedure for DTI processing was described previously (Xu et al., 2010). FMRIB’s Diffusion Toolbox (FDT) and Tract-Based Spatial Statistics (Smith, 2004; Smith et al., 2006, 2007) from FMRIB’s Software Library (FSL v5.0^1^; Smith et al., 2004; Woolrich et al., 2009) were used for image analyses. A set of mean images was created by aligning and averaging the two image sets from each subject and were used to construct the diffusion tensor using FDT. FDT typically generates maps of FA, λ_1_, MD, λ_2_, and λ_3_. The map of the perpendicular eigenvalue (λ_T_) was generated by averaging the maps of λ_2_ and λ_3_.

TBSS was used to register the FA map of each subject into Montreal Neurological Institute (MNI) template space. A mean FA map was created by averaging registered FA images from all subjects, and a mean FA skeleton was created by thinning the mean FA image (Smith, 2004; Smith et al., 2006, 2007). The aligned FA data of each participant were projected onto the mean skeleton by searching the area around the skeleton in the direction perpendicular to each tract, finding the highest local FA value, and assigning this value to the skeleton. The transformation matrices created for FA map registration were used to register λ_1_, λ_T_, and MD maps. Skeletons for λ_1_, λ_T_, and MD were generated using the same procedures for creating the FA skeleton.

The relationships between diffusion indices and quest subscale scores were assessed using whole-brain general linear regression models, created using GLM_setup in FSL. Whole-brain voxel-wise statistical testing was conducted using the “randomize” program with 5000 permutations. The “randomize” program uses permutation-based, non-parametric inferences to perform voxel-wise cross-subject statistics (Nichols and Holmes, 2002). Statistical thresholds for all image analyses were cluster *p* < 0.05 after family-wise-error (FWE) corrected for multiple comparisons using FSL’s threshold-free-cluster-enhancement (TFCE) algorithm. JHU ICBM-DTI-81 White-Matter Labels and JHU White-Matter Tractography Atlas provided by FSLVIEW (3.1) were used to identify the anatomical location of significant clusters in the brain (Mori et al., 2008, 2009). The function “fslmeants” from FSL was used to extract means of FA, λ_1_, λ_T_, and MD from each significant cluster surviving correction for multiple comparisons during whole-brain analysis. These means were used to demonstrate the relationships between DTI parameters and quest subscale scores using scatter plots, and no further statistical analyses were performed on these measures. A correction for multiple corrections beyond those used in DTI studies for whole-brain analyses (e.g., a Bonferroni correction for multiple comparisons involving three subscale measures and four DTI measures) was not employed.

## Results

**FIGURE 1:**
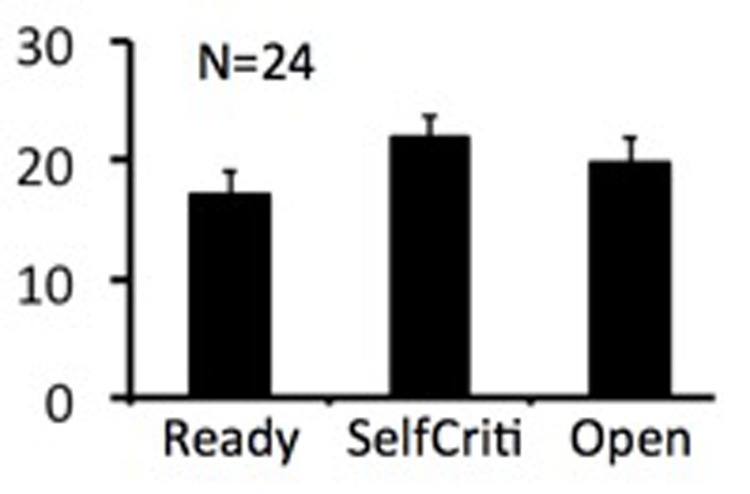
The bar graph shows the group mean scores on the three subscales of the quest scale. Error bars indicate standard errors (SEs) of the mean. “Ready” – Readiness to Face Existential Questions without Reducing their Complexity; “Doubt” – Self-Criticism and Perception of Religious Doubt as Positive; “Open” – Openness to Change Religious Beliefs.

**FIGURE 2:**
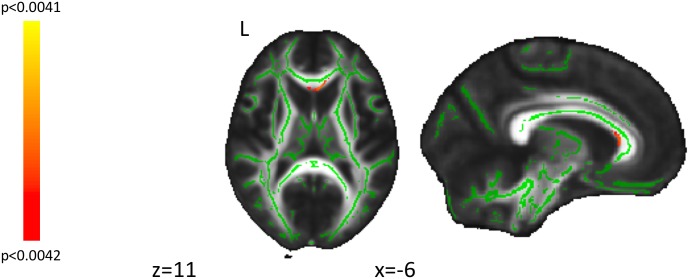
Displayed are findings from correlations of the quest openness-to-change subscale scores and perpendicular diffusion (λ_T_) values thresholded at *p*-value corrected using threshold-free cluster enhancement (*p*TFCE) at <0.0042. Significant voxels at a cluster threshold for whole-brain significance (*k* = 221) are shown in red. Green represents the center of white-matter tracts, as defined using the FMRIB58_FA_skeleton mask. L, left.

**FIGURE 3:**
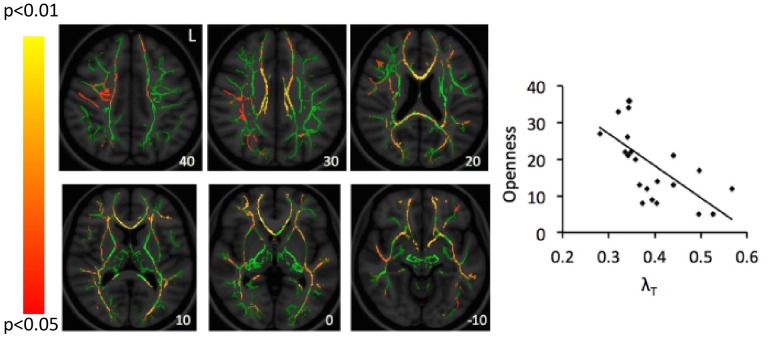
Correlations between scores on the openness-to-change measure and λ_T_ values. Red-yellow color on the Montreal Neurological Institute (MNI) T1 template indicates brain regions exhibiting significant correlations between scores of openness to change religious beliefs and λ_T_ values. For illustration, scatter-plots demonstrate correlations between scores of openness to change religious beliefs (Openness; *y*-axis) and mean λ_T_ values averaged across all clusters identified as significant in the whole-brain analysis. The number at the left bottom of each brain image indicates the *z* coordinate in the MNI space. L, left.

**FIGURE 4:**
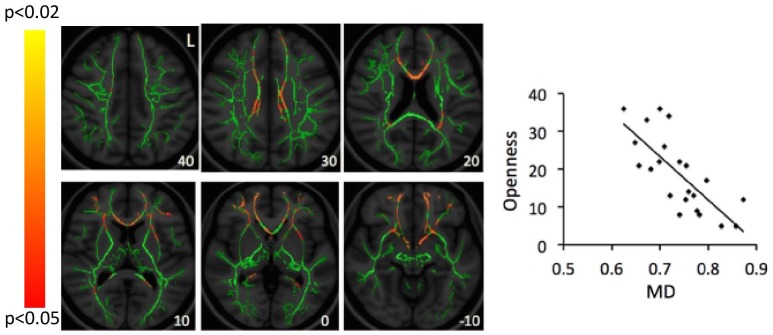
Correlations between scores on the openness-to-change measure and MD values. Red-yellow color on the Montreal Neurological Institute (MNI) T1 template indicates brain regions exhibiting significant correlations between scores of openness to change religious beliefs and MD values. For illustration, scatter-plots demonstrate correlations between scores of openness to change religious beliefs (Openness; *y*-axis) and mean MD values averaged across all clusters identified as significant in the whole-brain analysis. Number at the left bottom of each brain image indicates the *z* coordinate in the MNI space. L, left.

**Figure 1** shows the mean scores for the three subscales of the quest scale, with the openness-to-change scores being particularly relevant to the study hypothesis and subsequent figures. When applying a Bonferroni correction for 12 comparisons (3 subscales by 4 DTI parameters; *P* < 0.0042), a genual region of the corpus callosum was identified in the correlation between openness-to-change scores and λ_T_ values (**Figure 2**). When not applying a Bonferroni correction, scores on openness to change negatively correlated with λ_T_ and MD values in extensive white-matter regions in both hemispheres (**Figures 3, 4**). Regions showing significant negative correlations between scores on openness to change and λ_T_ values included the corpus callosum body, genu, and splenium, cingulum, SLF, anterior and superior CR, inferior fronto-occipital fasciculus, forceps minor, forceps major, external capsule, anterior limb of internal capsule, uncinate fasciculus, and sagittal stratum. Some, but not all, of these regions also showed significant negative correlations between scores on the openness-to-change subscale and MD. The implicated regions included the corpus callosum body, genu, and splenium, SLF, forceps minor, external capsule, and inferior fronto-occipital fasciculus. These significant correlations appeared driven by the λ_T_ values, because MD values represent the mean λ_T_ and λ_1_ values, and λ_1_ values did not show significant correlations with scores on the openness-to-change subscale. Scores on the openness-to-change subscale did not show significant correlations with FA values. Scores on the readiness-to-face-existential-questions and perception-of-religious-doubt-as-positive subscales did not show significant correlations with any DTI parameters.

## Discussion

To our knowledge, this study represents the first to investigate relationships between measures related to white-matter integrity and quest religious orientation. Our *a priori* hypothesis was partially supported as λ_T_ values correlated with openness-to-change scores and not with readiness-to-face-existential-questions or perception-of-religious-doubt-as-positive subscale scores. This finding was observed when correcting for whole-brain voxel-based tract-based spatial statistical analyses and when further applying a Bonferroni correction for 12 analyses involving 4 DTI and 3 quest subscale measures. When not applying a Bonferroni correction, λ_T_ and MD values in extensive regions of white matter were related to openness-to-change scores and not with readiness-to-face-existential-questions or perception-of-religious-doubt-as-positive subscale scores. Specifically, greater openness-to-change scores were related inversely to λ_T_ bilaterally in the corpus callosum body, genu, and splenium, cingulum, SLF, anterior and superior CR, inferior fronto-occipital fasciculus, forceps minor, forceps major, external capsule, anterior limb of internal capsule, uncinate fasciculus, and sagittal stratum. MD values in some but not all of these regions were also inversely to openness-to-change scores. Given that our *a priori* hypotheses were based on prior findings in a similar yet different area and concerns that stringent Bonferroni corrections may be ‘deleterious to sound statistical inference’ (Perneger, 1998, p. 1236), we will focus the discussion on non-Bonferroni-corrected findings while noting that the findings implicating the genu of the corpus callosum are the most statistically robust.

Despite the above-described correlations between the openness-to-change scores and λ_T_ and MD values, the former were not related to either λ1 or FA values. As diffusion-weighted MRI may be considered as indirect assessments of white-matter integrity, findings from these and other tensor-derived indices should be interpreted with caution. However, increases in both λ1 and FA have been linked to axonal loss, whereas increases in both MD and λ_T_ have been associated with demyelination (Song et al., 2002; Jones et al., 2013). The finding of significant negative correlations between λ_T_ and MD values and scores on openness to change suggests that better white-matter integrity in specific regions relates to a greater openness to change religious views. Different processes in the brain may influence λ_1_ and λ_T_ differently. Axonal degeneration has been associated with changes in λ_1_ value (Concha et al., 2006; Sidaros et al., 2008), while demyelination has been associated with increased λ_T_ values (Song et al., 2002). However, the current study investigated young adults who are unlikely to have experienced significant axonal degeneration or demyelination; thus, the findings are likely to reflect individual differences with respect to λ_T_, possibly relating to degrees of myelination. This line of reasoning suggests that greater openness to change religious beliefs may be related to better myelination of multiple white-matter tracts, and this may relate to better communication among brain regions connected by these tracts (Au Duong et al., 2005; Greicius et al., 2009). Future studies using different approaches (e.g., investigating how functional connectivity at rest or during cognitive and emotional processing relates to openness to change religious beliefs) are warranted in order to examine how openness to change religious beliefs may relate to other aspects of brain structure and function.

The corpus callosum body and forceps minor interconnect middle and superior frontal regions and motor, sensory, and auditory cortices, and are critical to interhemispheric communication (de Lacoste et al., 1985; Schulte et al., 2005; Voineskos et al., 2010). The SLF interconnects the PFC and the parietal cortex, is important for communication between these brain regions and contributes importantly to executive control, cognitive process, and emotional modulation (Alexander et al., 1986; Clark et al., 2009; Sexton et al., 2009). The inferior fronto-occipital fasciculus connects inferior and lateral margins of the occipital lobe to the lateral PFC and contributes importantly to the processing of visual and spatial information (Catani et al., 2002; Schmahmann et al., 2008). The present finding of correlations between measures of openness to change religious views and measures suggestive of better white-matter integrity in the corpus callosum, SLF, forceps minor, external capsule, and inferior fronto-occipital fasciculus is consistent with the notion that a greater openness to change religious views is associated with more efficient integration of information among extensive brain regions with wide-ranging functions in both hemispheres.

Changing and adapting one’s religious views as one grows and changes may be considered a creative process, as it involves integrating multiple domains of knowledge and employing divergent forms of thinking. Thus, it is worthwhile to consider whether these findings converge with previous brain research on creativity. Takeuchi et al. (2010) found that creativity correlated with measures linked to white-matter integrity in widespread bilateral regions which overlap with the current findings in the frontal lobe and corpus callosum body, as well as white-matter regions adjacent to the striatum, temporo-parietal junction, and inferior parietal lobe. In addition, creativity has been associated with greater interhemispheric and intrahemispheric EEG (electroencephalogram) findings (Jaušovec, 2000), right prefrontal cortex activation (Howard-Jones et al., 2005), and corpus callosum volume (Moore et al., 2009).

The findings suggestive of greater interhemispheric communication observed in the current data are noteworthy, as research suggests that the left and right hemispheres each possess particular specializations and encode information differently (McGlone, 1984; Barrett et al., 1998; Kosslyn et al., 1998; Heilman et al., 2000). Thus, increased interconnection between the two hemispheres may facilitate a variety of cognitive strategies to be used when negotiating and reconciling one’s religious views with other sources of knowledge and experience. Similarly, extensive white-matter connections throughout the brain may allow for the integration of knowledge previously encoded in disparate and/or isolated neural networks (Heilman et al., 2003). The current results are consistent with the notion that creativity involves efficient integration of information in the brain and facility with diverse cognitive functions (Takeuchi et al., 2010). It should be noted, however, that a previous DTI investigation on personality and creativity found negative correlations between FA values and both creativity and openness to experience in inferior frontal white matter (Jung et al., 2010), suggesting that these two tendencies may be related to poorer white-matter integrity. Further investigation could uncover nuances in relationships between creativity and white-matter integrity.

Research has furthermore suggested that post-traumatic growth directly correlates with both creative cognitive capacities and openness to change religious views (Calhoun et al., 2000; Peterson et al., 2008). Traumatic life events often upend people’s sense of self and view of the world (Calhoun et al., 2000). In addition, greater levels of religiosity have been reported following traumatic and stressful life events (Park et al., 1996). Thus, the capacity to embrace new religious perspectives may contribute to the capacity for resilience and for experiencing positive change in the aftermath of difficult life circumstances. Consistent with this notion, previous studies have linked reduced white-matter integrity with post-traumatic stress symptoms and greater white-matter integrity with increased resilience (Frodl et al., 2012; Daniels et al., 2013; Sanjuan et al., 2013; van der Werff et al., 2017).

The current study has several limitations. It identified correlations between measures related to white-matter integrity and aspects of religious quest, but the cross-sectional nature limits insight into causality. The study also relied on a relatively small sample of young adults. Thus, future studies should examine larger samples to investigate the extent to which the findings replicate and more diverse groups, including but not limited to older individuals. Additionally, possible gender-related differences should be examined. Ideally, future studies should also acquire a larger number of diffusion directions. While the different scalar indices used in this study each summarize different properties of the diffusion tensor and it has been recommended to use multiple indices in studies (Jones et al., 2013; Soares et al., 2013), they are not wholly independent. However, a Bonferroni correction for multiple corrections identified a region of the genu of the corpus callosum as most robustly related to λ_T_ values. In non-Bonferroni-corrected findings thresholded at *p*_TFCE_ < 0.05, the specificity of the findings to the subscale assessing openness to change religious views is in line with our *a priori* hypotheses based on our prior study of openness more broadly defined (Xu and Potenza, 2012). Additionally, the specificity of the relationship between openness to change religious views and both MD and λ_T_ and not λ_1_ or FA suggests a role for myelination in these relationships (Song et al., 2002; Jones et al., 2013), and this possibility should be examined in future studies. Furthermore, values of λ_1_ and λ_T_ are affected by crossing fibers, changes in fiber directions induced by local pathology (Wheeler-Kingshott and Cercignani, 2009; Jbabdi et al., 2010), and local neuropil packing density (Beaulieu, 2002). For these reasons, findings from the current study should be considered preliminary and reflective of measures that have been associated with white-matter integrity but may arise from multiple etiologies. Thus, interpretation is limited, and questions should be examined by other approaches.

## Conclusion

The current findings suggest that greater openness to changing religious views is related to better white-matter integrity in multiple tracts, including those connecting extensive brain regions between the two hemispheres. The widespread structural connections suggest neural pathways by which the capacity to embrace new religious perspectives as one grows and changes may occur. Furthermore, the results suggest brain structural features that may link openness to changing religious views to other potentially associated positive psychological tendencies, including those related to creative cognition and post-traumatic growth. Future studies should investigate these currently speculative possibilities, especially in larger and more diverse samples.

## Ethics Statement

This study was carried out in accordance with the recommendations of Yale Human Investigations Committee with written informed consent from all subjects. All subjects gave written informed consent in accordance with the Declaration of Helsinki. The protocol was approved by the Yale Human Investigations Committee.

## Author Contributions

LM and MP designed the study. IB acquired the data, and JX analyzed the data. CM and JX wrote the manuscript, while LM and MP revised the manuscript. All authors reviewed and approved the final manuscript.

## Conflict of Interest Statement

No funding agencies had input into the content of this manuscript, and none of the authors have any relevant conflicts of interests. MP has: consulted for Ironwood, Lundbeck, Shire, INSYS, RiverMend Health, Opiant/Lightlake Therapeutics, and Jazz Pharmaceuticals; received research support (to Yale) from Pfizer, Mohegan Sun Casino, and the National Center for Responsible Gaming; participated in surveys, mailings or telephone consultations related to addictions, impulse-control disorders or other health topics; consulted for and/or advised to gambling and legal entities on issues related to impulse-control/addictive disorders; provided clinical care in a problem gambling services program; performed grant reviews for the National Institutes of Health and other agencies; edited journals and journal sections; given academic lectures in grand rounds, CME events, and other clinical or scientific venues; and generated books or book chapters for publishers of mental health texts. The other authors declare that the research was conducted in the absence of any commercial or financial relationships that could be construed as a potential conflict of interest.

## Footnotes

1 http://www.fmrib.ox.ac.uk/fsl/g
